# Association between Sprint and Jump Performance and Maximum Strength in Standing Calf Raise or Squat in Elite Youth Soccer Players

**DOI:** 10.3390/sports12040087

**Published:** 2024-03-22

**Authors:** Björn Kadlubowski, Michael Keiner, Klaus Wirth, Robert Csapo

**Affiliations:** 1 Department of Sport and Human Movement Science, Centre for Sport Science and University Sports, University of Vienna, 1150 Vienna, Austria; robert.csapo@univie.ac.at; 2 Vienna Doctoral School of Pharmaceutical, Nutritional and Sport Sciences, University of Vienna, 1090 Vienna, Austria; 3 Department of Training and Exercise Science, German University of Health and Sport, 10587 Berlin, Germany; michaelkeiner@gmx.de; 4 Department of Training and Sport, University of Applied Sciences Wiener Neustadt, 2700 Wiener Neustadt, Austria; k.wirth@fhwn.ac.at

**Keywords:** plyometric, test, speed

## Abstract

Soccer is a complex sports discipline that requires players to engage in diverse high-intensity and multidirectional activities. The optimization of strength and conditioning programs requires a comprehensive understanding of the physical attributes influencing player performance. While previous research has demonstrated the influence of knee and hip extensor muscles on the performance in sprints and other explosive movements, this study aimed to establish the relationship between plantar flexor muscle strength and high-intensity actions. Back squat (BS) and calf raise (CR) one-repetition maxima as well as linear sprint (5-, 10-, 30 m) and drop jump performance from different heights (15, 30, 45 and 60 cm) were measured in 45 elite youth players (age: 16.62 ± 1.1 years). Results showed significant negative correlations between BS strength and sprint times (r = −0.60 to −0.61), confirming the importance of lower limb extensor muscle strength in short-distance sprints. While no significant correlations were found with sprint performances, CR strength was significantly associated with drop jump test results from 45 and 60 cm drop height (r = 0.36 to 0.46). These findings demonstrate that isolated CR strength positively influences the performance in actions involving rapid stretch-shortening cycles, which suggests that current strength and conditioning programs for youth soccer players should be extended to also include exercises specifically targeting the plantar flexor muscles. While this cross-sectional study provides novel insights into the complex interplay between muscle strength and soccer-specific performance, its findings need to be corroborated in longitudinal studies directly testing the impact of plantar flexor strength training.

## 1. Introduction

Soccer is a sports discipline characterized by a highly intermittent load structure. While a plethora of parameters, including technical and tactical factors, determine team performance, the physical demands of elite soccer players are high and have significantly increased in recent years [1]. In particular, high-intensity and multidirectional tasks, such as accelerations, decelerations, sprints, jumps or changes in direction are essential prerequisites for success. Match play analyses suggest that, in dependency of the playing position, soccer players perform between 20 and 39 sprints at velocities > 21 km/h [2] and may cover more than 1 km running at high speed or sprinting [3]. While the sprints account for only ~2–3% of the total distance covered in a match, it is the high-intensity efforts that mainly determine the outcome of matches. Indeed, more than 80% of all goals scored in the 2007/08 season of the German national league were preceded by a powerful action, such as straight sprints (45%), jumps (16%), rotations and change-in-direction sprints (each 6%) [4]. The importance of well-developed jumping abilities is further underlined by the observation that 44–56% of all goals scored in the Spanish and Italian soccer leagues are immediately preceded by headers [5]. Hence, muscle power generation in explosively performed actions is a critical performance-limiting factor, the development of which must be emphasized in youth players [6].

In an attempt to examine the determinants of performance in explosive actions, several studies have tested the correlations between parameters of fitness and the ability to sprint, jump and change direction. One of the earlier studies performed in young soccer players by Comfort et al. showed strong correlations between squat, sprint and jump performance [7]. These results have recently been confirmed by Keiner, Brauner, Kadlubowski, et al. [8] in elite youth players of various ages. Further studies investigated the associations between leg extension [9] or Nordic hamstring strength [10] and sprinting speed, as well as between the performance in the endurance plank and step 505 agility test [11]. Surprisingly, few studies performed to date have established the correlation between soccer players’ plantar flexor strength and performance in activities involving explosive muscle action. The strength of the plantar flexor muscles is critical for the performance in activities involving ankle joint stretch-shortening cycles (SSCs), such as hopping, skipping, jumping and sprinting. This is because the contractions of the plantar flexor muscles are expected to tauten the respective muscle–tendon units, thus benefitting the storage of elastic energy, and enhance the potential for contractile potentiation during the eccentric phase of movement; during the subsequent concentric phase, they would directly increase the propulsive impulse [12,13]. Despite this generally accepted understanding, there is limited research directly testing the influence of plantar flexor muscle strength on performance in SSC activities. One of the few correlational studies, which was performed in a sample of physical education students, reported moderate to strong correlations between calf raise strength and sprint performance [14].

The notion that the potential importance of the plantar flexor muscle strength may be a largely overlooked factor is also highlighted by the fact that training interventions aimed at improving the performance of young soccer players in high-intensity tasks typically rely on exercises aimed at strengthening the knee and hip extensor muscles [6,15,16,17,18,19,20], plyometric jumps or sprint training [21,22,23,24,25,26] or a combination of both [18,19,27,28,29,30,31]. By contrast, strength and conditioning programs for soccer players rarely include exercises to increase the maximum strength of the plantar flexors, such as calf raises [32,33], although maximum (isometric) strength and rapid force production are known to be correlated [34]. If a study were to reveal robust correlations between the maximal strength of the plantar flexor muscles and performance in high-intensity tasks, it would underscore the importance of prioritizing the enhancement of calf muscle strength within the training regimen of soccer players.

Considering the scarcity of data reflecting the association between maximum strength and performance in high-intensity, explosive tasks in youth soccer players, the present study aimed to investigate the relationship between the back squat and calf raise one-repetition maxima and the performance in linear sprints as well as countermovement, squat and drop jumps. It was hypothesized that both back squat and calf raise strength would be correlated with better sprint and jump performance.

## 2. Materials and Methods

### 2.1. Experimental Approach to the Problem

To test the above hypothesis, a cross-sectional study design was used. A sample of elite youth soccer players were tested for their one-repetition maxima (1-RM) in the back squat (BS) and standing calf raise (CR) exercise, linear sprint times over 5, 10 and 30 m as well as countermovement (CMJ), squat (SJ) and drop jump (DJ) performance from heights of 15, 30, 45 and 60 cm (DJ15, DJ30, DJ45, DJ60, respectively). The tests were performed on 2 test days separated by 2 days of recovery. One week prior to test day 1, the soccer players completed two preparatory sessions performed on separate days to familiarize with all the tests. On test day 1, the DJ, CMJ and SQ performance as well as the 1-RM BS were tested. On test day 2, the tests to assess linear sprint times and the 1-RM CR were performed.

### 2.2. Subjects

A total of 45 male youth soccer players (age: 16.62 ± 1.1 years old; height: 1.78 ± 0.06 m; weight: 67.7 ± 8.5 kg) were recruited from the under 17-years-old (U17, *n* = 44) and under 19-years-old (U19, *n* = 43) teams of one elite youth training center. Both youth soccer teams were engaged in the second highest league (U17/U19 Westfalenliga). The included soccer players participated in 4 training sessions per week and competed on weekends. All subjects had played soccer since early childhood and were not familiar with strength training. The training volume did not deviate between the teams. The subjects did not engage in fatiguing training sessions for a minimum of 3 days before testing. None of the subjects reported any injuries at the time of testing. All participants (and their parents, in the case of subjects who were younger than 18 years old) were informed of the experimental risks involved with the research before providing written informed consent to participate in this study. Approval for this study was obtained from the institutional review board at the German University of Health and Sport (DHGS-EK-2022-002). This study was performed in accordance with the Helsinki Declaration.

### 2.3. Measures and Procedures

Testing included the determination of the 1-RM BS (high bar). The barbell was positioned on the descending part of the trapezius muscle below the seventh cervical vertebra. The participants stood in an upright position with a self-selected distance between their feet, flexed their knees and hips to reach a deep squat position with proper form (top of thigh breaking parallel) and returned to the starting position. Attempts were considered invalid if the experienced examiner visually identified improper form, such as a rounding of the back or inadequate squat depth. A warm-up (2 sets of 6–8 repetitions) was performed with a submaximal, non-fatiguing load. The 1-RM was subsequently established through a series of maximally 5 trials, which were interspersed by at least 5 min of passive rest. The 1-RM BS is a highly reliable measure, with test-retest reliability intraclass correlation coefficients reportedly ranging between 0.91 and 0.99 [35]. Absolute maximum strength values were further normalized to body mass to determine relative maximum strength (Relative 1-RM BS).

The 1-RM CR test was performed on a standing calf raise machine (Flame Sport, Siauliai, Lithuania), which allows for a highly reliable test execution (test-retest reliability intraclass correlation coefficient of 0.91 (0.98–0.99)). Subjects were instructed to elevate their heels by plantar flexing the ankle joint; in agreement with previous studies [36,37], the range of motion was set to 4 cm, which was visually controlled through the use of a camera. Trials were considered invalid if participants failed to keep an upright posture, flexed their knees or were unable to plantar flex their ankles to raise their heels to the requested height. Proper execution was simultaneously controlled by 2 examiners. Just as for the assessment of the 1-RM BS, the actual test was preceded by a specific warm-up consisting of 2 sets of 6–8 repetitions performed with a submaximal, non-fatiguing load. Results were also normalized to body mass to yield relative maximum strength (Relative 1-RM CR).

The warm-up for the jump and sprint tests consisted of nonspecific running at low-to-medium intensity for approximately 5 min. Then, running coordination drills, including high knee skips and butt kicks (i.e., heel lift running), as well as side steps were performed for approximately 5 min. Subsequently, 3 acceleration runs over approximately 30 m were performed with short intervening walking breaks. Overall, the total warm-up time on each test day was 15 min. To assess sprint performance, each athlete was granted 3 attempts interspersed by 5 min breaks, and the 5, 10 and 30 m linear sprint time was measured via timing gates (Brower TC Timing System, Biederitz, Germany).

**Figure 1:**
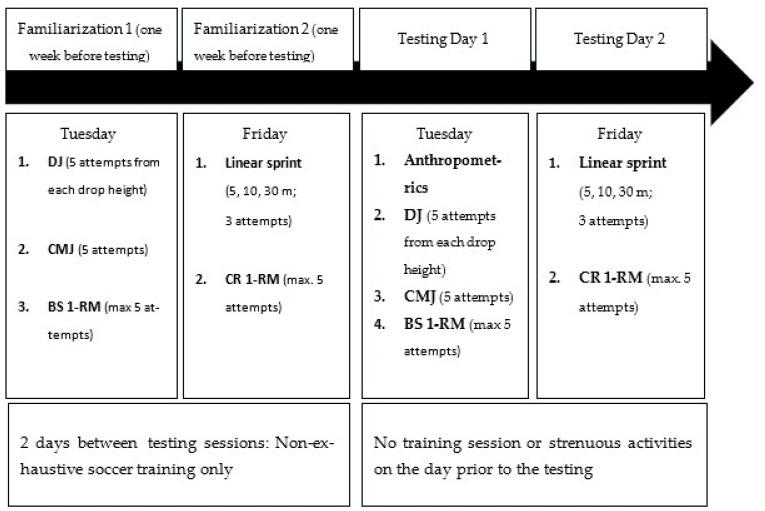
Sequence of tests and measurements performed before (T1) and after (T2) the training period. CMJ = Counter-Movement-Jump height; DJ = reactive strength index from 15/30/45/60 cm drop height, respectively; BS 1-RM = one-repetition maximum for the back squat; CR 1-RM = one-repetition maximum for the standing calf raise.

Jump performance was measured using a commercially available contact mat (Refitronic, Schmitten, Germany), which allows for jump height to be calculated from flight time (gt^2^/8; g = the gravitational acceleration [9.81 m·s^−2^] and t = flight time). The test-retest reliability for this system has been reported to be high, with intraclass correlation coefficients ranging between 0.85 and 0.93 [36,38]. The subjects performed 5 trials for each jump, and their best result was used for analysis. The athletes rested for 15 min between jumps of different kinds and for 1 min between individual attempts. The SJ was initiated at a knee angle of 90° without counter-movement and arm swings. The DJ was performed from different heights (15, 30, 45 and 60 cm). With their hands positioned above their hips, the participants were instructed to take a horizontal step from a box and jump as explosively and high as possible immediately after ground contact. To warrant minimal ground contact times (<250 ms), subjects were instructed to prevent their heels from touching the floor. A reactive strength index (RSI) was calculated as the ratio between jump height and ground contact time (RSI = jump height in cm/contact time in s) and interpreted as gross parameter indicative of DJ performance. Figure 1 shows a schematic diagram reflecting the sequence of measurements.

### 2.4. Analysis

The significance level for all statistical tests was set at *p* < 0.05. Normality of data was tested using the Kolmogorov–Smirnov test, and data were expressed as mean ± SD. Intraclass correlation coefficients (ICC) were calculated as measure of test-retest reliability and 95% confidence intervals (95% CI) were calculated for all variables. The ICC magnitudes were interpreted as follows: ICC < 0.5 = poor agreement, 0.5 ≤ ICC ≤ 0.75 = moderate agreement, 0.75 ≤ ICC ≤ 0.9 = good agreement, ICC ≥ 0.9 = excellent agreement. Pearson’s coefficient was calculated to establish correlations between the outcomes of the maximum strength tests (1-RM BS, 1-RM CS) and sprint and jump performance measures. The respective effect sizes were interpreted as per the following scale: 0 < r < 0.1 = very weak correlation, 0.1 ≤ r < 0.3 = weak correlation, 0.3 ≤ r < 0.5 = moderate correlation, 0.5 ≤ r < 0.7 = strong correlation, 0.7 ≤ r < 0.9 = very strong correlation, 0.9 ≤ r < 1.0 = nearly perfect correlation and perfect correlation [39]. A post hoc test of the correlation coefficients was made to determine the power of the results.

## 3. Results

The outcomes of speed and strength measurements as well as the respective test-retest reliability statistics are shown in Table 1. All parameters were normally distributed. In the drop jumps, the players consistently achieved ground contact times of <250 ms, irrespective of the drop height.

Correlational analyses showed a weak, yet statistically significant correlation between both the 1-RM CR and relative 1-RM CR and the drop jump performances from 45 and 60 cm (RSI 45 and RSI 60). Further significant and strong correlations were found between both the 1-RM BS and relative 1-RM BS and the 5, 10 and 30 m linear sprint performances. Also, weak correlations were observed between the relative 1-RM BS and the reactive strength indices RSI30 and RSI45. The results of the correlational analyses are shown in Table 2. A post hoc test showed that the statistical power of the correlation analyses ranged between 1–ß = 0.688 and 0.896 for the 1-RM CR and between 1–ß = 0.9945 and 0.999 for the 1-RM BS.

## 4. Discussion

The aim of the present study was to test the relationship between maximum strength in back squat and calf raise exercises and sprint and drop jump performances in youth elite soccer players. Our results showed significant correlations between 1-RM BS and sprint as well as between 1-RM CR and drop jump performance, providing insight into the relationship between the strength of muscle groups and performance in soccer. The 1-RM BS showed significant negative correlations with 5 m (r = −0.61), 10 m (r = −0.61), and 30 m (r = −0.60) sprint performances, implying that greater BS strength is associated with faster sprint times. These findings are in line with other studies to show moderate to high correlations between maximal strength and 5 m (r = −0.67 to −0.45) [7,14,40], as well as 10 m (r = −0.94 to −0.74) [14] and 30 m linear sprint performance (r = −0.73 to −0.6) [14,40] in soccer players.

In contrast to the findings of Keiner, Kadlubowski, Hartmann, et al. [37] and Möck, Hartmann, Wirth, et al. [14], which revealed a weak, yet significant correlation between 1-RM CR and 5 to 30 m sprint performance (r = −0.68 to −0.36), the present study did not uncover any significant associations between sprint performances and 1-RM CR. The absence of correlations between 1-RM CR and sprint performance in this study may be attributed to various contributing factors. Primarily, sprinting in soccer is a biomechanically intricate movement that engages a multitude of muscle groups, not limited to the calves. While calf strength certainly plays a role in generating power during sprinting, it represents only a partial facet of the comprehensive biomechanical processes involved. The complexities of the sport necessitate the coordinated action of various lower limb muscles, including the quadriceps, hamstrings and gluteal muscles [41] to propel players forward and maintain balance. Indeed, Pandy, Lai, Schache, et al. [42] estimated that the gastrocnemius muscle contributed only 37% of the total propulsive impulse during sprinting. The static and isolated nature of the calf raise exercise used for the calf strength assessment may not adequately capture the dynamic, multi-muscle actions integral to sprinting [43]. Furthermore, individual differences in running techniques, biomechanics and fitness levels among elite youth soccer players can contribute to the lack of a discernible correlation. It must also be acknowledged that sprinting performance can be significantly impacted by genetically predetermined factors, making them difficult to modify through physical training efforts [44]. Finally, it is important to note that the strength and conditioning programs of soccer players typically prioritize other physical attributes, such as agility, endurance or overall lower body strength, rather than calf-specific strength.

While no direct associations between calf strength and sprint performance were established, our study showed significant correlations between 1-RM CR (both in absolute terms and after normalization to body mass) and the RSIs obtained from the drop jump tests performed from higher drop heights (45, 60 cm), with correlation coefficients ranging from 0.36 to 0.46. The DJ performance from lower heights (15, 30 cm) showed no significant correlation between the 1-RM CR and the RSI (r = 0.09 to 0.16). It is worth mentioning that all correlations were observed alongside ground contact times below the fast SSC threshold of <250 ms. However, it is important to recognize that ground contact times during high-intensity actions, such as sprinting, may be even shorter [45]. One possible reason for the unique correlation between 1-RM CR and RSIs specifically from drops of 45 and 60 cm could be attributed to the increased requirement for force production during landing from greater elevations. Since higher drop heights impose greater eccentric loading on the muscles, individuals with stronger plantar flexors may better resist this eccentric load, leading to enhanced force generation during the subsequent concentric phase of the stretch-shortening cycle. In agreement with this notion, Möck, Hartmann, Wirth, et al. [14] proposed that increased maximum strength levels could potentially enable athletes to withstand and effectively utilize higher ground reaction forces. Further studies by Keiner, Kadlubowski, Hartmann, et al. [37] and Hartmann, Möck, Wirth, et al. [36] similarly observed correlations between the relative 1-RM CR and drop jump performance. Botero, Patiño Palma, Ramos-Parraci, et al. [46] emphasized the critical role of sprinting over extended distances, such as 50 m, in enhancing athletic performance and achieving success in soccer. Their research findings indicate that, among professional soccer players, proficiency in this type of sprint is closely linked to the capacity for performing rapid, repetitive jumps with short contact times, emphasizing the shared movement patterns between sprinting and reactive jumping in the context of professional soccer.

The RSIs derived from drop jump tests provide a valuable measure of an athlete’s capacity to effectively employ the SSC within their plantar flexor muscle–tendon units [47]. Leveraging the SSC allows athletes to seamlessly transition between the contrasting muscle actions involved in sprinting, leading to reduced ground contact times, enhanced force generation and the maintenance of higher running velocities through more efficient energy utilization [48,49]. Notably, increased RSIs are associated with heightened leg stiffness during maximum velocity running [50]. Leg stiffness is associated with muscle strength and strongly correlated with sprint speed [51,52]. Accordingly, stiffness is reportedly greater in trained sprinters compared to endurance runners [53]. Given this compelling body of evidence, it is entirely unsurprising that a recent systematic review reported moderate to strong correlations between reactive strength and pivotal components of sprint performance, including acceleration, top speed and change of direction speed [54]. In light of these findings, it is reasonable to postulate that, although our data did not reveal a direct correlation, augmenting the maximum strength of the plantar flexor muscles could indirectly enhance high-intensity actions, which are recognized as performance-limiting factors in soccer. This study’s robustness is influenced by its reliance on an ad hoc sample, specifically consisting of 45 soccer players aged between 16 and 19 years. While our sample is representative for elite youth soccer players, it is imperative to acknowledge that the results observed in such specific study cohorts may not be generalizable to other populations. The physiological, psychological and performance characteristics observed in this study may not be universally applicable to youth elite soccer players outside the 16 to 19 years age bracket or athletes participating in different sports.

## 5. Conclusions

In summary, this cross-sectional analysis provides valuable insights into the connections between maximum strength in squat and calf raise exercises, sprinting and drop jump performance in youth elite soccer players. Notably, our discovery of a significant correlation between 1-RM back squat strength and performance in 5, 10 and 30 m sprints underscores the pivotal role of lower limb extensor muscle strength in short-distance sprints. While we did not observe a direct correlation between maximum calf raise strength and sprint performance, we did find a positive association with drop jump performance from higher drop heights of 45 and 60 cm. This suggests that calf muscle strength could impact reactive force generation in stretch-shortening cycles, particularly when the eccentric loads acting on the muscle–tendon unit are high. Consequently, calf muscle strength might indirectly influence performance in high-intensity actions. Building upon this understanding, future longitudinal studies should aim to directly investigate the impact of various strength training interventions, incorporating either back squat, calf raise or plyometric training on the speed and jump performance of elite youth soccer players.

## Figures and Tables

**Table 1:** Mean, standard deviation, 95% confidence intervals and reliability statistics of all outcome variables.

Fitness Test	Mean ± SD	95% CI	ICC (95% CI)
5 m linear sprint (s)	0.98 (0.05)	0.97–1.00	0.897 (0.83–0.94)
10 m linear sprint (s)	1.70 (0.06)	1.68–1.72	0.935 (0.89–0.96)
30 m linear sprint (s)	4.14 (0.14)	4.10–4.18	0.972 (0.95–0.98)
CMJ (cm)	41.21 (4.53)	39.85–42.57	0.968 (0.95–0.98)
RSI 15 (cm/s)	156.82 (33.69)	150.54–170.05	0.943 (0.91–0.97)
RSI 30 (cm/s)	163.82 (39.99)	151.81–175.84	0.951 (0.92–0.97)
RSI 45 (cm/s)	163.24 (26.83)	155.18–171.30	0.847 (0.76–0.91)
RSI 60 (cm/s)	152.59 (36.54)	141.48–163.70	0.888 (0.80–0.94)
1-RM BS (kg)	94.09 (19.92)	88.03–100.15	0.989 (0.98–0.99)
1-RM CR (kg)	99.09 (21.10)	92.87–104.47	0.991 (0.98–0.99)
Relative 1-RM BS (kg/kg)	1.36 (0.22)	1.28–1.42	/
Relative 1-RM CR (kg/kg)	1.43 (0.32)	1.33–1.53	/

SD = Standard deviations; ICC = Intraclass correlation coefficient; 95% CI = Confidence intervals; CMJ = Counter-Movement-Jump height; RSI 15/30/45/60 = Reactive strength index from 15/30/45/60 cm drop height, respectively; 1-RM BS = Back squat one-repetition maximum; 1-RM CR = Standing calf raise one-repetition maximum; Relative 1-RM CR/Relative 1-RM BS = Back squat/Standing calf raise one-repetition maximum normalized to body mass.

**Table 2:** Correlation coefficient for the performance variables.

Variables	5 m Linear Sprint	10 m Linear Sprint	30 m Linear Sprint	CMJ	RSI15	RSI30	RSI45	RSI60
1-RM BS	−0.605 *	−0.613 *	−0.597 *	0.075	0.049	0.217	0.266	0.141
Relative 1-RM BS	−0.600 *	−0.641 *	−0.630 *	0.074	0.079	0.340 *	0.351 *	0.213
1-RM CR	−0.108	−0.083	−0.171	0.057	0.094	0.162	0.358 *	0.456 *
Relative 1-RM CR	0.028	0.018	−0.104	−0.074	0.101	0.197	0.348 *	0.456 *
5 m LS	/	/	/	−0.245 *	−0.243 *	−0.298 *	−0.264	−0.054
10 m LS	/	/	/	−0.293 *	−0.218 *	−0.225 *	−0.241	−0.057
30 m LS	/	/	/	−0.282 *	−0.092	−0.055	−0.126	−0.040

CMJ = Counter-Movement-Jump height; RSI 15/30/45/60 = Reactive strength index from 15/30/45/60 cm drop height, respectively; 1-RM BS = Back squat one-repetition maximum; 1-RM CR = Calf raise one-repetition maximum; Relative 1-RM CR/Relative 1-RM BS = Back squat/Standing calf raise one-repetition maximum normalized to body mass; * = significant at *p* < 0.05.

## Data Availability

The datasets generated and/or analyzed in connection with the present investigation can be obtained from the corresponding author upon reasonable request.

## References

[1] Bradley P., Archer D., Hogg R., Schuth G., Bush M., Carling C., Barnes C. (2015). Tier-Specific Evolution of Match Performance Characteristics in the English Premier League: It’s Getting Tougher at the Top. J. Sports Sci..

[2] Ade J., Fitzpatrick J., Bradley P. (2016). High-Intensity Efforts in Elite Soccer Matches and Associated Movement Patterns, Technical Skills and Tactical Actions. Information for Position-Specific Training Drills. J. Sports Sci..

[3] Di Salvo V., Pigozzi F., Gonzalez-Haro C., Laughlin M., De Witt J. (2012). Match Performance Comparison in Top English Soccer Leagues. Int. J. Sports Med..

[4] Faude O., Koch T., Meyer T. (2012). Straight sprinting is the most frequent action in goal situations in professional football. J. Sports Sci..

[5] Sciberras C., Attard K., Muscat-Inglott M., Kerr-Cumbo R. (2022). An Analysis of Goals Scored in the Maltese Football Premier League 2018–2019. MCAST J. Appl. Res. Pract..

[6] Sander A., Keiner M., Wirth K., Schmidtbleicher D. (2013). Influence of a 2-year strength training programme on power performance in elite youth soccer players. Eur. J. Sport. Sci..

[7] Comfort P., Stewart A., Bloom L., Clarkson B. (2013). Relationships Between Strength, Sprint, and Jump Performance in Well-Trained Youth Soccer Players. J. Strength Cond. Res..

[8] Keiner M., Brauner T., Kadlubowski B., Sander A., Wirth K. (2022). The Influence of Maximum Squatting Strength on Jump and Sprint Performance: A Cross-Sectional Analysis of 492 Youth Soccer Players. Int. J. Environ. Res..

[9] Peñailillo L., Espildora F., Jannas-Vela S., Mujika I., Zbinden-Foncea H. (2016). Muscle Strength and Speed Performance in Youth Soccer Players. J. Hum. Kinet..

[10] Markovic G., Sarabon N., Boban F., Zorić I., Jelcic M., Šoš K., Scappaticci M. (2018). Nordic Hamstring Strength of Highly Trained Youth Football Players and Its Relation to Sprint Performance. J. Strength Cond. Res..

[11] Imai A., Kaneoka K. (2016). The relationship between trunk endurance plank tests and athletic performance tests in adolscent soccer players. Int. J. Sports Phys. Ther..

[12] Cormie P., McGuigan M., Newton R. (2010). Changes in the Eccentric Phase Contribute to Improved Stretch-Shorten Cycle Performance after Training. Med. Sci. Sports Exerc..

[13] Radnor J., Oliver J., Waugh C., Myer G., Moore I., Lloyd R. (2018). The Influence of Growth and Maturation on Stretch-Shortening Cycle Function in Youth. Sports Med..

[14] Möck S., Hartmann R., Wirth K., Rosenkranz G., Mickel C. (2018). Correlation of dynamic strength in the standing calf raise with sprinting performance in consecutive sections up to 30 meters. Res. Sports Med..

[15] Chelly M.S., Fadhloun M., Cherif N., Amar M., Tabka Z., Van Praagh E. (2009). Effects of a Back Squat Training Program on Leg Power, Jump, and Sprint Performances in Junior Soccer Players. J. Strength Cond. Res..

[16] Keiner M., Sander A., Wirth K., Schmidtbleicher D. (2013). Long-Term Strength Training Effects on Change-of-Direction Sprint Performance. J. Strength Cond. Res..

[17] Styles W., Matthews M., Comfort P. (2015). Effects of Strength Training on Squat and Sprint Performance in Soccer Players. J. Strength Cond. Res..

[18] Hammami M., Negra Y., Shephard R., Chelly M.S. (2017). The effect of standard strength- vs. contrast strength training on the development of sprint, agility, repeated change of direction and jump in male junior soccer players. J. Strength Cond. Res..

[19] Ferley D., Scholten S., Vukovich M. (2020). Combined Sprint Interval, Plyometric, and Strength Training in Adolescent Soccer Players: Effects on Measures of Speed, Strength, Power, Change of Direction, and Anaerobic Capacity. J. Strength Cond. Res..

[20] Wong D.P., Chamari K., Wisloff U. (2010). Effects of 12-Week On-Field Combined Strength and Power Training on Physical Performance Among U-14 Young Soccer Players. J. Strength Cond. Res..

[21] Buchheit M., Mendez-Villanueva A., Delhomel G., Brughelli M., Ahmaidi S. (2010). Improving Repeated Sprint Ability in Young Elite Soccer Players: Repeated Shuttle Sprints Vs. Explosive Strength Training. J. Strength Cond. Res..

[22] Sáez de Villarreal E., Suarez-Arrones L., Requena B., Haff G., Ferrete Cáceres C. (2015). Effects of Plyometric and Sprint Training on Physical and Technical Skill Performance in Adolescent Soccer Players. J. Strength Cond. Res..

[23] Beato M., Bianchi M., Coratella G., Merlini M., Drust B. (2017). Effects of Plyometric and Directional Training on Speed and Jump Performance in Elite Youth Soccer Players. J. Strength Cond. Res..

[24] Makhlouf I., Chaouachi A., Chaouachi M., Granacher U., Behm D. (2018). Combination of Agility and Plyometric Training Provides Similar Training Benefits as Combined Balance and Plyometric Training in Young Soccer Players. Front. Physiol..

[25] Negra Y., Chaabene H., Fernandez-Fernandez J., Sammoud S., Bouguezzi R., Prieske O., Granacher U. (2018). Short-Term Plyometric Jump Training Improves Repeated-Sprint Ability in Prepuberal Male Soccer Players. J. Strength Cond. Res..

[26] Derakhti M., Bremec D., Kambič T., Siethoff L., Psilander N. (2021). Four Weeks of Power Optimized Sprint Training Improves Sprint Performance in Adolescent Soccer Players. Int. J. Sports Physiol. Perform..

[27] de Hoyo M., Gonzalo-Skok O., Sanudo B., Carrascal C., Plaza-Armas J., Camacho-Candil F., Otero-Esquina C. (2016). Comparative Effects of In-Season Full-Back Squat, Resisted Sprint Training, and Plyometric Training on Explosive Performance in U-19 Elite Soccer Players. J. Strength Cond. Res..

[28] Spineti J., Figueiredo T., Oliveira V., Barbosa M., Oliveira L., Miranda H., Reis V., Simão R. (2015). Comparison between traditional strength training and complex contrast training on repeated-shuttle-sprint ability and muscle architecture in male elite soccer players. J. Sports Med. Phys. Fit..

[29] Giovanni F., Mariano I., Iuliano E., Giombini A., Ciccarelli A., Buonsenso A., Calcagno G., di Cagno A. (2020). Isoinertial Eccentric-Overload Training in Young Soccer Players: Effects on Strength, Sprint, Change of Direction, Agility and Soccer Shooting Precision. J. Sports Sci. Med..

[30] Keiner M., Kadlubowski B., Sander A., Hartmann H., Wirth K. (2020). Effects of 10 months Speed, Functional, and Traditional Strength Training on Strength, Linear Sprint, Change of Direction, and Jump Performance in Trained Adolescent Soccer Players. J. Strength Cond. Res..

[31] Gorostiaga E., Izquierdo M., Ruesta M., Iribarren J., González-Badillo J., Ibáñez J. (2004). Strength training effects on physical performance and serum hormones in young soccer players. Eur. J. Appl. Physiol..

[32] Siegler J., Gaskill S., Ruby B. (2003). Changes Evaluated in Soccer-Specific Power Endurance Either With or Without a 10-Week, In-Season, Intermittent, High-Intensity Training Protocol. J. Strength Cond. Res..

[33] Suarez-Arrones L., Sáez de Villarreal E., Nuñez F., Di Salvo V., Petri C., Buccolini A., Maldonado R., Torreño N., Mendez-Villanueva A. (2018). In-season eccentric-overload training in elite soccer players: Effects on body composition, strength and sprint performance. PLoS ONE.

[34] Peterson M., Alvar B., Rhea M. (2006). The Contribution of Maximal Force Production to Explosive Movement Among Young Collegiate Athletes. J. Strength. Cond. Res..

[35] McMaster D., Gill N., Cronin J., McGuigan M. (2014). A Brief Review of Strength and Ballistic Assessment Methodologies in Sport. Sports Med..

[36] Hartmann R., Möck S., Wirth K., Mickel C. (2017). Zum Zusammenhang zwischen dem dynamischen Kraftmaximum der Plantarflexoren mit der Reaktivkraftleistung im Drop Jump aus verschiedenen Fallhöhen. Leipz. Sportwiss. Beiträge: Jahrg..

[37] Keiner M., Kadlubowski B., Hartmann H., Stefer T., Wirth K. (2021). The influence of maximum strength performance in squats and standing calf raises on squat jumps, drop jumps, and linear as well as change of direction sprint performance in youth soccer athletes. Int. J. Sports Exerc. Med..

[38] Keiner M., Sander A., Hartmann H., Mickel C., Wirth K. (2018). Do long-term strength training and age affect the performance of drop jump in adolescents?. J. Strength Cond. Res..

[39] Shrout P.E., Fleiss J.L. (1979). Intraclass correlations: Uses in assessing rater reliability. Psychol. Bull..

[40] McBride J., Blow D., Kirby T.J., Haines T., Dayne A., Triplett N. (2009). Relationship Between Maximal Squat Strength and Five, Ten-, and Forty-Yard Sprint Times. J. Strength Cond. Res..

[41] Howard R., Conway R., Harrison A. (2017). Muscle activity in sprinting: A review. Sports Biomech..

[42] Pandy M., Lai A., Schache A., Lin Y.-C. (2021). How muscles maximize performance in accelerated sprinting. Scand. J. Med. Sci. Sports.

[43] Bolger R., Kenny I., Lyons M., Harrison A. (2014). Sprinting Performance and Resistance Based Training Interventions: A systematic review with meta-analysis. J. Strength Cond. Res..

[44] Majumdar A., Robergs R. (2011). The Science of Speed: Determinants of Performance in the 100 m Sprint: A Response to Commentary. Int. J. Sports Sci. Coach..

[45] Blauberger P., Horsch A., Lames M. (2021). Detection of Ground Contact Times with Inertial Sensors in Elite 100-m Sprints under Competitive Field Conditions. Sensors.

[46] Botero C., Patiño Palma B., Ramos-Parraci C., Gómez Rodas A., Afanador-Restrepo D., Claros J. (2023). Vertical jump performance and the relationship with sprint speed at 20 m and 50 m in professional soccer players. F1000Research.

[47] Suchomel T., Sole C., Stone M. (2015). Comparison of Methods That Assess Lower-body Stretch-Shortening Cycle Utilization. J. Strength Cond. Res..

[48] Douglas J., Pearson S., Ross A., McGuigan M. (2017). Kinetic Determinants of Reactive Strength in Highly Trained Sprint Athletes. J. Strength Cond. Res..

[49] Takahashi K., Shirai Y., Oki S., Nabekura Y. (2021). The effect of a decrease in stretch-shortening cycle function after cycling on subsequent running. J. Sci. Med. Sport.

[50] Douglas J., Pearson S., Ross A., McGuigan M. (2019). Reactive and Eccentric Strength Contribute to Stiffness Regulation during Maximum Velocity Sprinting in Team Sport Athletes and Highly Trained Sprinters. J. Sports Sci..

[51] Meyers R., Moeskops S., Oliver J., Hughes M., Cronin J., Lloyd R. (2017). Lower-Limb Stiffness and Maximal Sprint Speed in 11–16-Year-Old Boys. J. Strength Cond. Res..

[52] Secomb J., Nimphius S., Farley O., Lundgren L., Tran T., Sheppard J. (2015). Relationships Between Lower-Body Muscle Structure and, Lower-Body Strength, Explosiveness and Eccentric Leg Stiffness in Adolescent Athletes. J. Sports Sci. Med..

[53] Harrison A., Keane S., Coglan J. (2004). Force-Velocity Relationship and Stretch-Shortening Cycle Function in Sprint and Endurance Athletes. J. Strength Cond. Res..

[54] Jarvis P., Turner A., Read P., Bishop C. (2022). Reactive Strength Index and its Associations to Measures of Physical and Sports Performance: A Systematic Review with Meta-Analysis. Sports Med..

